# Rationale and Design of the CREDENCE Trial: computed TomogRaphic evaluation of atherosclerotic DEtermiNants of myocardial IsChEmia

**DOI:** 10.1186/s12872-016-0360-x

**Published:** 2016-10-06

**Authors:** Asim Rizvi, Bríain ó. Hartaigh, Paul Knaapen, Jonathon Leipsic, Leslee J. Shaw, Daniele Andreini, Gianluca Pontone, Subha Raman, Muhammad Akram Khan, Michael Ridner, Faisal Nabi, Alessia Gimelli, James Jang, Jason Cole, Ryo Nakazato, Christopher Zarins, Donghee Han, Ji Hyun Lee, Jackie Szymonifika, Millie J. Gomez, Quynh A. Truong, Hyuk-Jae Chang, Fay Y. Lin, James K. Min

**Affiliations:** 1 Dalio Institute of Cardiovascular Imaging, NewYork-Presbyterian Hospital, New York, NY USA; 2 Department of Cardiology, VU University Medical Center, Amsterdam, The Netherlands; 3 Department of Medical Imaging, University of British Columbia, St. Paul’s Hospital, Vancouver, BC Canada; 4 Emory University School of Medicine, Atlanta, GA USA; 5 Centro Cardiologico Monzino, IRCCS, Milan, Italy; 6 Istituto di Ricovero e Cura a Carattere Scientifico, Centro Cardiologico Monzino, Milan, Italy; 7 Davis Heart and Lung Research Institute and Heart Center, The Ohio State University, Columbus, OH USA; 8 The Cardiac Center of Texas, McKinney, TX USA; 9 Heart Center Research, LLC, Huntsville, AL USA; 10 Houston Methodist DeBakey Heart & Vascular Center, Houston Methodist Hospital, Houston, TX USA; 11 Fondazione Toscana/CNR Gabriele Monasterio, Pisa, Italy; 12 Division of Cardiology, Kaiser Permanente San Jose Medical Center, San Jose, CA USA; 13 Mobile Cardiology Associates, Mobile, AL USA; 14 Cardiovascular Imaging Lab, St. Luke’s International Hospital, Tokyo, Japan; 15 Heart Flow, Inc., Redwood City, CA USA; 16 Department of Surgery, Stanford University, Stanford, CA USA; 17 Department of Medicine, Weill Cornell Medical College, New York, NY USA; 18 Department of Radiology, Weill Cornell Medical College, New York, NY USA; 19 Division of Cardiology, Severance Cardiovascular Hospital, Yonsei University Health System, Seoul, South Korea; 20 Radiology and Medicine, New York-Presbyterian Hospital and Weill Cornell Medical College, 413 E. 69th Street, Suite 108, New York, NY 10021 USA

**Keywords:** Coronary computed tomography angiography, Coronary artery disease, Fractional flow reserve, Myocardial blood flow, Myocardial perfusion scintigraphy

## Abstract

**Background:**

Coronary computed tomography angiography (CCTA) allows for non-invasive assessment of obstructive coronary artery disease (CAD) beyond measures of stenosis severity alone. This assessment includes atherosclerotic plaque characteristics (APCs) and calculation of fractional flow reserve (FFR) from CCTA (FFR_CT_). Similarly, stress imaging by myocardial perfusion scintigraphy (MPS) provides vital information. To date, the diagnostic performance of integrated CCTA assessment versus integrated MPS assessment for diagnosis of vessel-specific ischemia remains underexplored.

**Methods:**

CREDENCE will enroll adult individuals with symptoms suspicious of CAD referred for non-emergent invasive coronary angiography (ICA), but without known CAD. All participants will undergo CCTA, MPS, ICA and FFR. FFR will be performed for lesions identified at the time of ICA to be ≥40 and <90 % stenosis, or those clinically indicated for evaluation. Study analyses will focus on diagnostic performance of CCTA versus MPS against invasive FFR reference standard. An integrated stenosis-APC-FFR_CT_ metric by CCTA for vessel-specific ischemia will be developed from derivation cohort and tested against a validation cohort. Similarly, integrated metric by MPS for vessel-specific ischemia will be developed, validated and compared. An FFR value of ≤0.80 will be considered as ischemia causing. The primary endpoint will be the diagnostic accuracy of vessel territory-specific ischemia of integrated stenosis-APC-FFR_CT_ measure by CCTA, compared with perfusion or perfusion–myocardial blood flow stress imaging testing, against invasive FFR.

**Discussion:**

CREDENCE will determine the performance of integrated CCTA metric compared to integrated MPS measure for diagnosis of vessel-specific ischemia. If proven successful, this study may reduce the number of missed diagnoses and help to optimally predict ischemia-causing lesions.

**Trial registration:**

ClinicalTrials.gov, NCT02173275. Registered on June 23, 2014.

**Electronic supplementary material:**

The online version of this article (doi:10.1186/s12872-016-0360-x) contains supplementary material, which is available to authorized users.

## Background

Coronary revascularization remains a mainstay of treatment for coronary artery disease (CAD), which continues to affect more than 16 million US adults. Functional myocardial ischemia may result from anatomically obstructive CAD and strongly determines prognosis, and thus is useful for guiding decisions of revascularization [1, 2]. Invasive fractional flow reserve (FFR) is the reference standard for determining the physiologic significance of CAD for vessel-specific ischemia among patients with multi-vessel CAD. However, the invasive nature of FFR measurement limits this evaluation to only high-risk patients with indications for ICA.

Until now, for the vast numbers of patients at low or intermediate risk for CAD, numerous non-invasive stress imaging modalities including echocardiography, cardiac magnetic resonance (CMR), and myocardial perfusion scintigraphy (MPS) have served as a mainstay for identifying ischemia by detecting stress-induced regional myocardial perfusion defects, of which the most commonly employed is MPS [3, 4]. At a per-patient level, MPS by single photon emission computed tomography (SPECT) or positron emission tomography (PET) is capable of determining the severity and extent of myocardial ischemia with high performance [5, 6]. However, the ability of MPS to correctly discriminate ischemia on a per-vessel basis is less robust [7]. Further, the accuracy of MPS is compromised in patients with multi-vessel CAD [8]. These findings therefore raise questions regarding the ability of MPS to correctly identify coronary lesions that would benefit from revascularization [9].

In the recent past, coronary computed tomography angiography (CCTA) has emerged as a promising non-invasive imaging modality for the diagnosis of anatomically obstructive CAD, with high sensitivity and negative predictive value compared to ICA [10, 11]. Although CCTA enables discrimination of stenosis with high accuracy, it can overestimate stenosis, and similar to ICA, may misidentify ischemia [12]. These findings have led to the emergence of several methods using CCTA-specific data beyond luminal stenosis severity alone for the diagnosis of lesion-specific ischemia. These methods include additional atherosclerotic plaque characteristics (APCs) and calculation of FFR from CCTA (FFR_CT_).

APCs assessment by CCTA has demonstrated high agreement with invasive methods of plaque assessment and has shown to improve discrimination of ischemia-causing coronary lesions [13]. These include plaque burden and composition, and arterial remodeling [14, 15]. Previous studies have reported that selected APCs are also associated with ischemia by MPS [16, 17]. Yet, the entirety of APCs to optimize the precise identification of ischemia-causing culprit lesions has not been examined to date.

FFR_CT_ represents a novel non-invasive imaging technique that can evaluate the physiologic significance of CAD and enables the calculation of vessel-specific FFR [18]. Further still, given the computational modeling technique in FFR_CT_ calculations, it is possible to place a “virtual stent” by modeling the resolution of luminal compromise. In a recent prospective multicenter study, FFR_CT_ was found to be superior to CCTA stenosis alone for diagnosis of ischemia-causing lesions, and demonstrated good correlation with FFR [19].

Similar to CCTA, which can provide important information beyond luminal stenosis severity alone, stress imaging testing can also do so by an array of prognostically and diagnostically important factors. These include high-risk imaging features (e.g., transient ischemic dilation, stress-induced lung uptake of radiotracer, increased right ventricular uptake), myocardial blood flow (MBF), exercise electrocardiographic (ECG) findings, functional capacity, stress-induced symptom complexes. Even within these categories, other more granular data are available, e.g., in ECG data, extent of ST-segment depression, rate-pressure product, heart rate recovery, and other variables are well known to augment CAD diagnosis.

The overall objective of the CREDENCE trial is to determine the utility of a novel diagnostic integrated approach by integrating anatomic APCs with physiologic FFR_CT_ to optimize the precise identification of vessel-specific ischemia by CCTA, and to compare this measure against the totality of relevant variables by MPS.

## Methods/Design

The Computed TomogRaphic Evaluation of Atherosclerotic DEtermiNants of Myocardial IsChEmia (CREDENCE) trial (clinicaltrials.gov NCT02173275) is a prospective multicenter cross-sectional study of 618 individuals (*n* = 309 [derivation cohort]; *n* = 309 [validation cohort]) wherein eligible participants will undergo CCTA, MPS, ICA and FFR. For the purposes of the study, either MPS, CCTA, and/or FFR will have been performed for clinical purposes, with the other test being performed as part of the trial procedure. Study analyses will focus on the diagnostic performance of MPS versus CCTA against the gold standard of invasively determined FFR for vessel-specific ischemia.

**Fig. 1:**
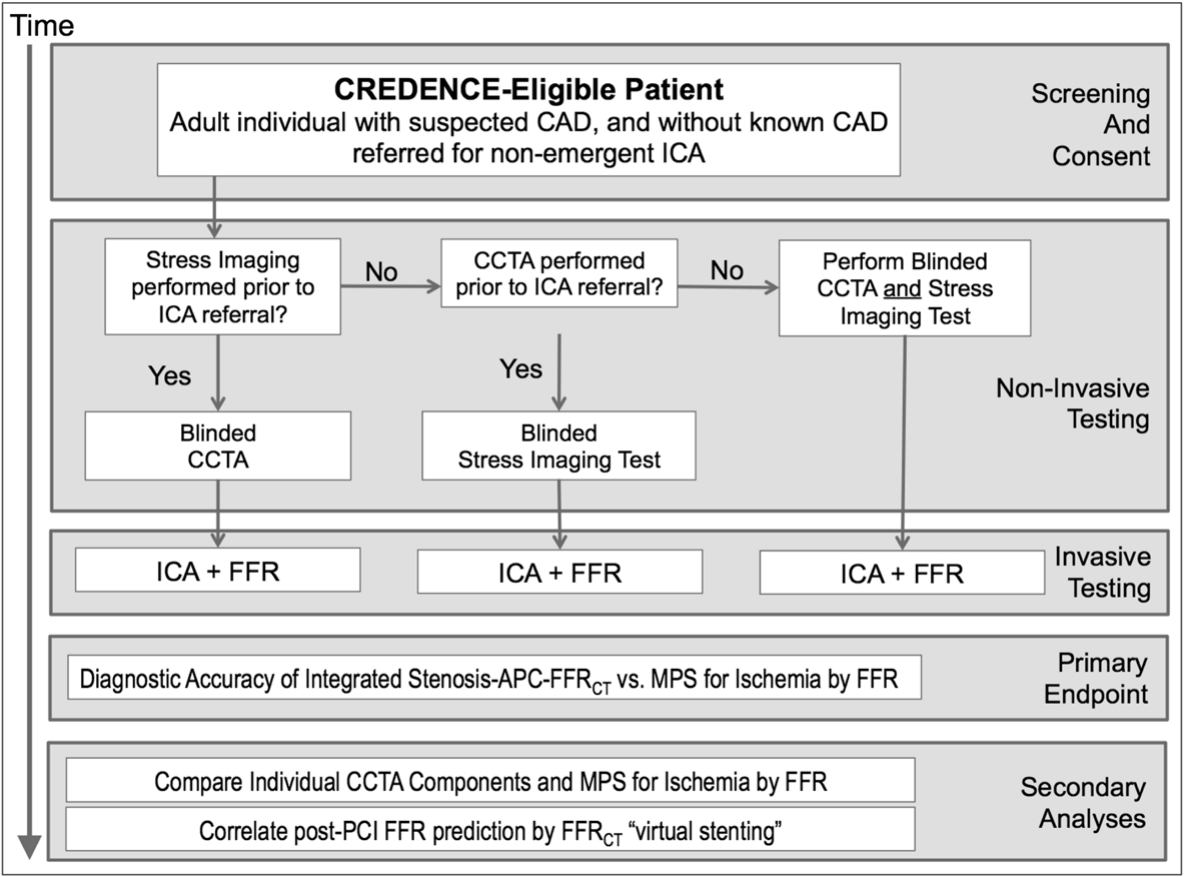
CREDENCE study design. CAD, coronary artery disease; ICA, invasive coronary angiography; CCTA, coronary computed tomography angiography; MPS, myocardial perfusion scintigraphy; FFR, fractional flow reserve; FFR_CT_, fractional flow reserve derived from coronary computed tomography angiography; APCs, atherosclerotic plaque characteristics; PCI, percutaneous coronary intervention

The relationships of anatomic APCs and physiologic FFR_CT_ by CCTA will be assessed according to measures of vessel-specific ischemia, referenced to an invasive FFR standard. An integrated anatomic-physiologic metric by CCTA for vessel-specific ischemia will be developed from a derivation cohort and tested against a validation cohort, both of which will undergo CCTA, MPS, ICA and FFR. Similarly, an integrated metric by MPS for vessel-specific ischemia will be developed and validated. The performance of this integrated CCTA measure will be directly compared with MPS for diagnosis of vessels that manifest ischemia. The schematic summary of the trial design is depicted in Fig. 1.

Importantly, for reasons of safety, all FFRs to be performed will be limited to lesions identified at the time of ICA to be ≥40 and <90 % stenosis, or those that should be clinically indicated for evaluation. As lesions <40 % in diameter stenosis severity are almost uniformly non-ischemic and those ≥90 % are typically ischemic, these lesions should be avoided for interrogation with an FFR guidewire for reasons of patient safety, unless deemed medically necessary. In contrast, coronary stenoses between 40 and 90 % demonstrate variable rates of ischemia and may be appropriately interrogated for clinical indications. If FFR interrogation of a coronary lesion between 40 and 90 % is considered as imparting excess risk to a study subject for anatomical or other reasons, it will be avoided. In keeping with prior studies, vessel territories will be comprised of the left anterior descending artery [LAD] (and diagonal branches), the left circumflex artery [LCx] (and obtuse marginal branches), and the right coronary artery [RCA] (and posterolateral branch and posterior descending artery). Patients who undergo percutaneous coronary intervention (PCI) by intracoronary stenting of ≥1 coronary lesions after the ICA and FFR measurements may have repeat measurements of FFR after the completion of stent deployment, as clinically indicated.

The CREDENCE study will be performed in 19 investigative sites in USA, Canada, Netherlands, Japan, China, Latvia, Italy, and South Korea (ClinicalTrials.gov, NCT02173275). Each center may not enroll more than 40 % of the total number of subjects. The shortest time duration between noninvasive and invasive testing will be at the discretion of the local physician. The maximum time permitted between noninvasive and invasive tests will be 60 days.

## Study objectives

### Primary objective

The primary objective is to determine the diagnostic accuracy of vessel territory-specific ischemia by use of an integrated stenosis-APC-FFR_CT_ measure determined by CCTA, when compared with perfusion or perfusion–MBF stress imaging testing, against invasive FFR. Stress imaging will also include additional characteristics, as described above, which do not exhibit co-linearity with other variables. CCTA and stress imaging features will be hierarchically evaluated: CCTA will be evaluated by stenosis, then FFR_CT_, then APCs; while stress imaging will be evaluated by perfusion, then MBF, then ECG features and finally, functional capacity.

### Secondary objectives

The secondary objective is to compare the accuracy of the individual components of the CCTA and MPS measures to detect ischemia by invasive FFR: APC components; FFR_CT_; MPS vessel-specific perfusion deficits; and reduced MBF. Another objective is to determine the accuracy of FFR_CT_ “virtual stenting” with a post-PCI FFR value of >0.80 in an effort to assess the correlation between FFR_CT_ “virtual stenting” to post-PCI FFR.

## Target population

The CREDENCE target population will comprise a large and representative sample of individuals for whom CCTA and MPS testing confer the largest potential benefit; that is, for those with suspected CAD who are being referred for non-emergent clinically indicated ICA based upon an imaging study (either MPS or CCTA). In this regard, CREDENCE will enroll adult individuals who meet the inclusion criteria and none of the exclusion criteria, as detailed in Table 1. The study is considered non-significant risk because all subjects will undergo clinically-indicated ICA as planned, with FFR performed in vessel territories with >40 % stenosis for which there is equipoise of ischemia presence [20].

**Table 1:** CREDENCE trial inclusion and exclusion criteria

*Inclusion criteria*	
(1) Age ≥18 years	
(2) Patients scheduled to undergo clinically-indicated non-emergent invasive coronary angiography	
*Exclusion criteria*	
(1) Known coronary artery disease (myocardial infarction, percutaneous coronary interventions, coronary artery bypass graft)	
(2) Hemodynamic instability	
(3) Inability to provide written informed consent	
(4) Concomitant participation in another clinical trial in which an individual is subject to investigational drug or device	
(5) Pregnancy or unknown pregnancy status	
(6) Absolute contraindication to iodinated contrast due to prior near-fatal anaphylactoid reaction (laryngospasm, bronchospasm, cardiorespiratory collapse, or equivalent)	
(7) Impaired chronic renal function (serum creatinine ≥1.7 mg/dl or Glomerular Filtration Rate <30 ml/min)	
(8) Baseline irregular heart rhythm (e.g., atrial fibrillation, etc.)	
(9) Heart rate ≥100 beats per minute	
(10) Systolic blood pressure ≤90 mm Hg	
(11) Contraindications to β blockers or nitroglycerin or adenosine	

## Efficacy analyses and sample size

### Primary efficacy analysis

The primary statistical analysis will be diagnostic accuracy of vessel territory-specific ischemia using an integrated APC-FFR_CT_ metric by CCTA, as compared with territory-specific ischemia on perfusion or perfusion-MBF stress imaging, against invasive FFR. Three vessel territories including the LAD, LCx, and RCA will be examined. The non-ischemic vessels will serve as controls to ischemic vessels. An FFR measurement cutoff value of ≤0.80 will be considered as ischemia-causing, and >0.80 as non ischemia-causing.

### Analysis of APCs and FFR_CT_ against an invasive FFR standard

APCs and FFR_CT_ will be assessed against an invasive FFR standard for diagnosis of lesion-specific ischemia. Direct comparison of an integrated APC-FFR_CT_ metric to a non-invasive gold standard by a composite perfusion, MBF, ECG, and functional capacity metric will then be made.

### (A) Assessment of APCs for diagnosis of lesion-specific ischemia

Coronary vessels from 309 patients (927 vessels) with moderate or severe ischemia by MPS and who undergo ICA will be studied, so as to determine the physiologically most relevant APCs. If there is no ischemia in a vessel, then the angiographically most severe lesion measured by invasive FFR will be chosen for APC assessment. For these vessels, associations of the APC measures will be examined in relation to vessel-based ischemia.

### Within-subject comparison of APCs of ischemia-causing versus non ischemia-causing coronary lesions

APCs within ischemia-causing lesions will be compared with within-subject control lesions. Matched analysis will be performed to predict the likelihood of a vessel being ischemic based on APCs for lesions (e.g., matched 1:1 within persons); with an ischemic vessel––that possess the angiographically most severe stenosis and is measured by invasive FFR––matched to one of the remaining arteries (LAD, LCx or RCA).

Moreover, additional ischemic:nonischemic vessel combinations will be considered. For any patient with one ischemic vessel, an analysis will be performed using a 1:2 case:control match to compare results with the 1:1 matched analysis. For individuals with two ischemic vessels, a 2:1 case:control match will be performed for each ischemic vessel to the same non-ischemic control vessel, and then comparing these results with the 1:1 matched analysis.

### (B) Assessment of FFR_CT_ for diagnosis of lesion-specific ischemia

In individuals who experience ischemia, coronary lesions from 309 patients will be studied, and ischemic vessels will be identified by invasive FFR. The region within the same coronary vessel will be identified distal to the most angiographically obstructive stenosis on the CCTA image that was measured by FFR. The agreement between measured FFR_CT_ and invasive FFR will be examined for these vessels, both as dichotomous (≤ versus >0.80) and continuous (ranging from 0 to 1) variables. In addition, the diagnostic performance of FFR_CT_ to invasive FFR will be tested by measures of sensitivity, specificity, positive predictive value (PPV), and negative predictive value (NPV).

### Within-subject comparison of FFR_CT_ of ischemia-causing lesions to non-ischemia causing coronary lesions

FFR_CT_ for ischemic vessels will be compared with within-subject controls. Matched analysis will be performed to predict the likelihood of a vessel to be ischemic based on FFR_CT_ for lesions matched 1:1 within a person. Within-subject matched comparisons will be performed as discussed earlier for the APCs.

### (C) Derivation and validation of an integrated anatomic-physiologic CCTA metric for diagnosis of ischemia

An integrated CCTA metric will then be developed that will represent the most parsimonious set of APCs for improving diagnosis of vessel-specific ischemia. The weighted APC set will be combined to FFR_CT_ values using information derived from (A) and (B). Multivariate mixed-effects logistic regression analyses will be conducted with APC-FFR_CT_ measures as a predictor of ischemia. A backward stepwise selection method will be used so that only significant variables at *p* < 0.05 level will be included in the final model. Since these analyses are on a per-vessel basis, a random effects model will be used to account for within-patient clustering. Finally, a summary APC-FFR_CT_ metric as a predictor of ischemia, accounting for the weighted contribution of APCs and FFR_CT_, will be obtained. This summary APC-FFR_CT_ metric will be tested for its ability to diagnose vessel-based ischemia in a validation cohort of 309 patients who will also undergo CCTA, MPS, ICA, and FFR.

### (D) Comparison of an integrated anatomic-physiologic CCTA metric to vessel-territory specific ischemia by MPS

In the validation cohort, the integrated APC-FFR_CT_ metric will be directly compared against MPS for the diagnosis of vessel-specific ischemia, using invasive FFR as a reference standard. This integrated metric will be compared against a combined perfusion-MBF-ECG-functional capacity measure by MPS so as to assure the highest diagnostic performance of both CCTA and MPS in comparison. MBF is anticipated to be most useful in the presence of balanced reduction of perfusion (i.e., in the absence of relative perfusion differences) and thus, is expected to increase specificity when a regional vessel-based territory appears normal by perfusion.

### Secondary efficacy analysis

The secondary statistical analysis will compare the accuracy of the individual components of the CCTA and MPS measures to detect ischemia by invasive FFR: APC components; FFR_CT;_ MPS vessel-specific perfusion deficits; and reduced MBF. Another analysis will compare the accuracy of FFR_CT_ “virtual stenting” with a post-PCI FFR value of >0.80, and to determine the correlation between the FFR_CT_ “virtual stenting” and post-PCI FFR. For segments that were treated with coronary stent and underwent FFR, the post-PCI FFR value will be measured. On a per-segment analysis, “virtual stenting” will be performed and post-“virtual stent” FFR_CT_ will be predicted. The diagnostic accuracy of FFR_CT_ “virtual stenting” to post-PCI FFR will then be calculated, using a cutoff value of >0.80 to be considered non-ischemic and a successful intervention. Further, the correlation between post-FFR_CT_ “virtual stenting” and post-PCI FFR will be performed using Pearson’s correlation or Spearman’s rank correlation, as appropriate.

## Sample size calculation and statistical power

A total of 618 participants will be enrolled in this study. The sample size determination of the primary endpoint was based upon a sequential derivation-validation design for APCs and FFR_CT_. For a logistic regression of a binary response variable, a sample size of 618 patients is needed for a 90 % power at a 0.05 significance level to detect a 30 % increase in the probability that a vessel manifest ischemia. This change corresponds to an odds ratio of 2.0. The study expects to have 309 patients available that will provide more than sufficient sample for variation in the estimated parameters for sample size. To ensure statistical validity, a post hoc power analysis will be run and the actual power achieved by the analysis will be reported.

## Quality assurance

### CCTA image acquisition and interpretation

All CCTA scans will be performed with single- or dual source CCTA scanners with a minimum of 64 detector rows. Detector row width will be ≤0.75 mm. At a minimum, single-head power injectors allowing fast injection rates of 4–7 mL/s will be required. Laboratories will follow local CCTA scanning protocols as long as they are consistent with quality standards defined by the Society of Cardiovascular Computed Tomography (SCCT) guidelines [21]. CCTA interpretation has also been detailed elsewhere, and will conform to SCCT guidelines [21].

CCTA images will be transmitted to independent masked readers at the CCTA Core Laboratory, who will use dedicated 3-dimensional workstations to evaluate the CCTA images. The 18-segment SCCT model of the coronary tree will be used for the coronary evaluation [21]. Coronary atherosclerosis will be defined as any tissue >1 mm^2^ within or adjacent to the lumen that can be discriminated from surrounding pericardial tissue, epicardial fat, or lumen; and identified in ≥2 planes. Stenoses will be graded in accordance with the SCCT segmental 5-point scale: 0 %; 1–24 %; 25–49 %, 50–69 %, and 70–100 % [21]. Plaque will be measured on 3days workstations, with reconstructions at the smallest slice thickness (~500 μm). All CCTA scans will be interpreted by a minimum of 2 readers, with 20 % randomly repeated to test for inter- and intra-observer variability. APCs and FFR_CT_ analyses will be performed using the already-acquired CCTA data set. CCTA core lab readers and automated software will perform interpretation of APCs. APCs will be studied by multiplanar reconstructions (MPR) and cross-sections using optimal phases, and by automated software with manual correction, with segments classified by an 18-segment SCCT model [21].

### Atherosclerotic plaque characteristics

Given their association to myocardial ischemia in prior studies, six coronary APCs will be selected and will include 1) low attenuation plaque (LAP); 2) positive remodeling (PR); 3) mixed plaque (MP); 4) plaque burden (PB); 5) minimal luminal area (MLA); and 6) transluminal attenuation gradient (TAG) [12, 16, 17, 22]. To determine the incremental value of APCs beyond conventional CCTA measures of CAD, baseline CAD burden by the CAD index and CAD complexity by the SYNTAX score will also be determined [23].

Briefly, a Hounsfield unit (HU) <30 will signify LAP, as this threshold exhibits the best performance compared to intravascular ultrasound (IVUS) [15]. A remodeling index (RI) >1.10 will be defined as PR and a RI <0.95 will be defined as negative remodeling, with in-between values categorized as intermediate remodeling [13]. Plaque composition will be classified on a 5-level scale: non-calcified (0 % calcified plaque [CP]), mostly non-calcified (1–30 % CP), mixed (30–70 % CP), mostly calcified (70–99 % CP), and calcified (100 % CP). Plaque composition scores will be made by summing the number of segments with each type of plaque composition. Finally, TAG will be defined as the change in HU per mm of artery, and will be calculated from the coefficient of linear regression between luminal attenuation and length from vessel ostium [24]. An automated plaque analysis will also be performed with a validated software (QAngio CT, Medis medical imaging systems, Leiden, the Netherlands).

### FFR_CT_ and virtual stenting

FFR_CT_ will be performed by applying a computational fluid dynamics (CFD) approach to typically-acquired CCTA scans for calculation of coronary artery pressure and flow by HeartFlow, Inc. (Redwood City, CA). More detailed information regarding FFR_CT_ and virtual stenting is provided in the online supplementary material, Additional file 1.

### Myocardial perfusion scintigraphy

MPS will be performed by a variety of methods, including PET, SPECT, or CMR. Standard acquisition and analysis protocols will be agreed on for each technique covering patient preparation, cardiovascular stress, administration of radiopharmaceutical or contrast medium, image acquisition and quality control, image processing and interpretation. These procedures will be based on available international guidelines [25, 26].

As such, all MPS studies in this investigation will employ PET when possible. Briefly, dynamic MPS will be performed at rest and during vasodilator stress by 13 N-ammonia (13-NH3) or rubidium-82 (Rb-82) PET using a high-resolution PET/CT scanner, in accordance with societal guidelines [27]. Data will be reconstructed with attenuation correction, based on a validated model from our lab to quantify radionuclide signal attenuation and peak absolute MBF as well as MBF reserve in the territory of the LAD, LCx, and RCA. Time-activity curves will be constructed beginning with initiation of 13-N-NH3 or Rb-82 infusion, using spillover correction [28, 29]. Radionuclide signal in each territory from stress and rest will be compared for calculation of regional (per-vessel) total perfusion defect (rTPD, amount of hypoperfused myocardium as % of total myocardium). Stress-induced perfusion in each territory will be defined as [stress rTPD – rest rTPD], or “reversible” rTPD (REVrTPD), which will be examined continuously for regional myocardial ischemia [30]. In cases when PET cannot be performed, MPS by SPECT will be done and will evaluate perfusion as described above.

For MPS, perfusion in each of the 17 segments will be classified as normal, mild reduction, moderate reduction, severe reduction, or absent perfusion, and the segmental scores were summed for the stress and rest images. An inducible perfusion abnormality will be defined as a summed segmental difference score between stress and rest images ≥2, either from a score ≥1 in ≥2 contiguous segments, or ≥2 in ≥1 segment. Scarring will be defined similarly from the summed segmental rest score. For wall motion abnormalities (WMA), segmental myocardial wall motion will be scored at rest and during stress as normal, hypokinetic, akinetic, or dyskinetic. Inducible ischemia will be defined as an increase in segmental wall motion score ≥1 from rest to stress in ≥2 contiguous segments. Scarring will be defined similarly from the resting wall motion score [31]. The angiographic dominance of the vascular territories will be used for designation of territories during MPS as previously described [32, 33]. In patients with left coronary dominance, the inferior and infero-lateral segments will be assigned to the LCx territory, and all 4 apical segments will be assigned to the LAD territory [33]. In the case of RCA dominance, the inferior, infero-lateral, and apical inferior segments will be assigned to the RCA territory, with the remaining 3 apical segments assigned to the LAD territory [33].

### Invasive coronary angiography and fractional flow reserve

Patients will undergo diagnostic ICA in accordance with usual clinical indications and by imaging standards set forth by the American College of Cardiology/Society for Cardiac Angiography and Interventions [34]. Further detailed information about ICA and FFR procedure is outlined in the online supplementary material, Additional file 1.

### Integration of FFR, CCTA and FFR_CT_ measurements

For suitable comparison of CCTA APCs, FFR_CT_, and FFR, accurate alignment of CCTA and ICA must be performed. An integration process will be performed in a masked fashion by a study investigator not responsible for CCTA, FFR_CT_, or MPS evaluation in order to identify the location of a lesion by CCTA that corresponds to the location where the distal pressure sensor of the wire is located at the time of FFR. The latter integration approach will ensure complete masking of the interpretation of CCTA APCs, FFR_CT_, and ICA and FFR results. On this model, the cardiologist will demarcate the location of the FFR wire, and will refer the model to investigators responsible for interpretation of the CCTA APCs and FFR_CT_.

## Organization and data management

This study will be coordinated by the Dalio Institute of Cardiovascular Imaging Clinical and Data Coordinating Center (DICI CDCC). The DICI CDCC will be responsible for the processing and quality control of the data. The study protocol will be approved at each participating center by the local institutional review board. Completed electronic case report forms (eCRFs) will be entered by trained site personnel, and will be transmitted to a central data repository at the DCC. At regular intervals, all data will be transferred from electronic case report form (REDCAP) to SAS for statistical summarization, data description, and data analysis. Further cross-checking of the data will be performed in SAS.

Site qualification will be issued by the DICI CDCC for each modality before subject enrollment. Before beginning enrollment, eligible sites will be qualified by the imaging Core Laboratories based on site surveys and on successful transfer of one or more complete datasets with sufficient image quality and completeness for each modality. During the study, technical quality assessment of image and test acquisition will be accomplished on all studies by central repository research technicians trained by the CDCC. This ongoing review will ensure the adequate quality and completeness of datasets.

## Discussion

The CREDENCE study aims to integrate the anatomic and physiologic information derived from CCTA for the precise identification of ischemia-causing coronary lesions, and to compare this measure against an integrated metric for MPS. To date, no non-invasive imaging modality has been routinely capable of a “one-stop shop,” wherein anatomic identification of stenoses and physiologic determination of ischemia are derived from a single test, thus resulting in layered testing and increased complexity and cost of clinical care. Specifically, this study will assess the performance of anatomic APCs by CCTA and physiologic FFR_CT_ for diagnosis of vessel-specific ischemia. An integrated APC-FFR_CT_ metric by CCTA will then be directly compared to a perfusion-MBF-ECG-functional capacity metric by MPS for diagnosis of vessel-specific ischemia. As such, we hypothesize that an integrated assessment by CCTA will be superior to an integrated assessment by MPS for the diagnosis of vessel-specific ischemia.

Prior studies have established that MPS is capable of determining the severity and extent of myocardial ischemia with high performance at a per-patient level, and that MPS evaluation is augmented by MBF, ECG, and functional capacity findings [5, 6]. Nevertheless, the “real world” accuracy of MPS is less assertive despite its high reported diagnostic performance [35]. To date, no study has included the totality of information imparted by MPS—including MBF, ECG, and functional capacity—rendering comparisons of CCTA to MPS incomplete and, in general, skewed towards improved CCTA diagnostic performance.

CCTA has demonstrated high performance for diagnosis of obstructive CAD compared to ICA, and predicts adverse prognosis [36]. In the prospective multicenter ACCURACY trial, CCTA demonstrated a sensitivity, specificity, PPV and NPV of 94, 83, 48, and 99 %, respectively, compared with ICA. The results of this ACCURACY trial are in agreement with two subsequent multicenter studies that demonstrated high accuracy of CCTA [10, 11]. On the contrary, even though CCTA can discriminate stenosis with high accuracy, the ability to identify ischemia-causing coronary lesions is suboptimal at present [12]. In a study of 79 patients undergoing CCTA and ICA with FFR, high rates of false positive CCTAs were noted, with an overall specificity for ischemia of 40 % [12]. Similar findings have been observed from pooled analyses comparing CCTA to MPS wherein CCTA stenoses were associated with ischemia less than half of the time [37, 38].

The PROMISE trial demonstrated equivalent outcomes between functional imaging by multiple modalities, including PET, and anatomic evaluation of stenosis severity by CCTA [39]. The CREDENCE trial posits that stenosis evaluation alone discards important anatomic and functional data in routinely performed CCTA, and that a combined assessment may be superior for vessel ischemia. Similarly, the CREDENCE trial posits that perfusion evaluation alone by MPS discards important functional information, and that combined assessment may be superior for vessel ischemia. Prior studies have shown that CCTA enables assessment of several coronary APCs with generally high accuracy beyond luminal diameter stenosis, and similar to invasive studies by intravascular ultrasound, APCs visualized on CCTA have been associated with ischemia-causing culprit lesions [40] [14, 15, 17]. Further still, the accuracy of FFR_CT_ for diagnosing coronary ischemia has been reported in several studies [19, 41, 42]. In the DISCOVER-FLOW (Diagnosis of Ischemia-Causing Stenoses Obtained Via Noninvasive Fractional Flow Reserve) trial, compared with invasive FFR, non-invasive FFR_CT_ demonstrated per-vessel accuracy, sensitivity, specificity, PPV, and NPV for ischemia-causing lesions of 84.3 %, 87.9 %, 82.2 %, 73.9 %, 92.2 %, respectively [19]. Further, in a more recent HeartFlow NXT study, the FFR_CT_ data matched closely with invasively measured FFR [42].

To date, however, clinical reporting of CCTA has been limited to stenosis severity, with relative neglect of APCs and unavailability of FFR_CT_. The aversion to incorporate APC findings may originate from the fact that APC quantification is time-intensive with per-patient evaluation taking 8–10 h for comprehensive APC evaluation. This study will facilitate widespread applicability by assessing co-linearity of APCs to develop the most parsimonious set of APCs that offers the greatest diagnostic value; and validate the diagnostic utility of the APCs in a separate cohort of individuals. We have ensured that APC measures will be accurate by several safeguards. We will use images only from CCTA scanners with 64-rows or more, the threshold for adequate coronary imaging by CCTA. Further, APCs will be interpreted at a state-of-the-art laboratory by ≥2 readers with inter- and intra-observer assessment for a 20 % of CCTAs for assurances of reliability. Similarly, this study will assess FFR_CT_, referenced against both invasive FFR and non-invasive stress test “gold” standards. Furthermore, this study will perform APC and FFR_CT_ evaluation with automated software that will facilitate time-efficient evaluation of both APCs and FFR_CT_.

Similar to CCTA, we will perform a comprehensive assessment of MPS. By a dedicated and blinded core laboratory, we will examine factors beyond regional myocardial perfusion, and will include imaging high-risk features of CAD (including transient ischemic dilatation, and increased lung uptake of radiotracer), as well as high-risk ECG features (such as ST-segment depression, hypotensive response to stress, heart rate recovery, and rate-pressure product). Functional capacity and MBF will also be assessed.

If proven successful and the superiority of a combined anatomic-physiologic approach is shown, this trial will provide the rationale in developing and establishing a novel diagnostic paradigm that not only identifies patients who manifest ischemia, but also specify ischemia-causing coronary lesions that may be more accurate than conventional stress imaging. If the strategies are equivalent, there will still be information gain in determining the most efficient means to determine lesion-specific ischemia by CCTA and MPS. The results of this approach may reduce the number of missed diagnoses and help to optimally predict ischemia-causing lesions, considering the fact that millions of patients are tested for CAD each year in the US alone. Further, this novel diagnostic paradigm may help promote avoidance of unnecessary invasive procedures and procedure-related complications in the future, and allow for improved clinical decision-making to identify patients who are versus who are not eligible for coronary revascularization. Finally, the proposed trial will potentially reduce downstream care related to these invasive procedures as well as the cost burden, and directly impacts annual healthcare savings.

## New knowledge gained

The CREDENCE study aims to fill many gaps in knowledge and is distinctive from other prior studies in determining which coronary vessels can be non-invasively determined as manifesting ischemia. By comparing an integrated CCTA measure directly against an integrated measure for MPS, this study will provide unavailable comparative effectiveness data for CCTA and stress testing for diagnosis of lesion-specific ischemia. Moreover, the proposed trial will potentially provide improved patient care and may directly impact clinical practice by reducing rates of unnecessary invasive procedures as well as health care costs.

## Additional file

Additional file 1: Online supplementary material. (DOCX 23 kb)

## Abbreviations

13-NH3: 13 N-ammonia
APCs: Atherosclerotic plaque characteristics
CAD: Coronary artery disease
CCTA: Coronary computed tomography angiography
CFD: Computational fluid dynamics
CMR: Cardiac magnetic resonance
CREDENCE: Computed TomogRaphic Evaluation of Atherosclerotic DEtermiNants of Myocardial IsChEmia
DICI CDCC: Dalio Institute of Cardiovascular Imaging Clinical and Data Coordinating Center
DISCOVER-FLOW: Diagnosis of Ischemia-causing stenoses obtained via noninvasive fractional flow reserve
ECG: Electrocardiography
eCRFs: Electronic case report forms
FFR: Fractional flow reserve
FFR_CT_: Fractional flow reserve from coronary computed tomography angiography
HU: Hounsfield unit
ICA: Invasive coronary angiography
IVUS: Intravascular ultrasound
LAD: Left anterior descending artery
LAP: Low attenuation plaque
LCx: Left circumflex artery
MBF: Myocardial blood flow
MLA: Minimal luminal area
MP: Mixed plaque
MPR: Multiplanar reconstructions
MPS: Myocardial perfusion scintigraphy
PB: Plaque burden
PCI: Percutaneous coronary intervention
PET: Positron emission tomography
PR: Positive remodeling
Rb-82: Rubidium-82
RCA: Right coronary artery
REVrTPD: Reversible regional total perfusion defect
RI: Remodeling index
rTPD: Regional total perfusion defect
SCCT: Society of Cardiovascular Computed Tomography
SPECT: Single photon emission computed tomography
TAG: Transluminal attenuation gradient
WMA: Wall motion abnormalities
