# Supplementary material for: Rationale and Design of the CREDENCE Trial: computed TomogRaphic evaluation of atherosclerotic DEtermiNants of myocardial IsChEmia
Source: BMC Cardiovasc Disord. 2016 Oct 6;16:190. doi: 10.1186/s12872-016-0360-x (PMC5053174; doi:10.1186/s12872-016-0360-x)
Supplement: Supplementary file 1 — Online supplementary material. (DOCX 23 kb) [file 12872_2016_360_MOESM1_ESM.docx]

**Additional file 1. Online Supplementary Material**

**FFR_CT_ and Virtual Stenting**

Three-dimensional blood flow simulations in the coronary arteries will be performed in a masked fashion with proprietary software on site at HeartFlow, Inc. (Redwood City, CA). The patient anatomy from a mid-diastolic time point will be modeled because this time point usually corresponds to the best imaging resolution. The simulations utilize a 3D model of the coronary arteries extracted from CCTA data and separate models representing the heart, distal coronary vasculature, and downstream aortic conditions. Model parameters will be assigned by customizing coronary physiology data to each patient-specific model. To enable comparison with pressure and flow data obtained during diagnostic cardiac catheterization, coronary blood flow will be simulated under conditions of adenosine-mediated hyperemia. The simulation will compute pressure and flow in the coronary arteries. The FFR_CT_ will be obtained by dividing the mean pressure distal to the coronary stenosis by the mean aortic pressure. Accuracy of FFR_CT_ will be determined by comparison with measured FFR during ICA.

The pre-stent computational model will be marked with the location of the stent used to treat the patient. Analysts will then perform virtual coronary intervention by modifying the computational model in the region of the stent to enlarge the lumen of the treated coronary segment according to the proximal and distal reference areas. Analysts will be masked to the results of the treatment and to all FFR measurements. Computational analysis of hyperemic coronary flow and pressure for the entire heart model will be repeated to determine post-PCI FFR_CT_. A point on the computational model will be selected that match the location of post-PCI FFR measurement to determine the corresponding FFR_CT_ value beyond that stenosis.

**Invasive Coronary Angiography**

Patients will undergo diagnostic ICA by board-certified interventional cardiologists or country-equivalent in accordance with usual clinical indications and by imaging standards set forth by the American College of Cardiology/Society for Cardiac Angiography and Interventions [1]. 100-200 mcg intracoronary nitroglycerin will be sequentially administered in the left and right coronary arteries before initial cineangiograms, unless contraindicated. Coronary arterial images must be obtained with selective catheterization of left and right coronary arteries. ICA images will be transmitted to independent masked readers at the Angiographic Core Laboratory.

Similar to CCTA images, an 18-segmental model of the coronary tree will be used for coronary evaluation [2]. ICA will be analyzed by validated quantitative coronary angiography software, employing the use of automated edge-detection algorithms. Employing the outer diameter of the coronary injection catheter as a standard for calibration, a minimum lumen diameter and percentage stenosis will be measured from the view that demonstrates the greatest reduction in luminal diameter with the least amount of foreshortening of the segment at a motion-free state during the cardiac cycle (typically during diastole). The stenosis will represent a relative reduction in comparison to the most normal appearing region proximal and distal to the stenosis. All vessels ≥1.5 mm in diameter will be measured. If a total occlusion is observed, all segments distal to that occlusion will not be assessed.

Fractional Flow Reserve

Experienced interventional cardiologists will perform the FFR procedure. FFR will be obtained using a sensor-tipped 0.014-inch guidewire (PressureWire, St. Jude Medical, St. Paul, MN or PrimeWire, Volcano, San Diego, CA). Within each artery, the guidewire will be introduced into the vessel that demonstrates the greatest stenosis and is >2 mm in diameter, with FFR performed for each artery during maximal hyperemia. Maximal myocardial hyperemia will be induced by continuous intravenous infusion of adenosine in a central vein at an infusion rate of 140 mcg/kg per min for a minimum of 2 minutes.

During maximum hyperemia, FFR will be calculated as the ratio of distal mean pressure measured (Pd) by the pressure wire divided by the mean proximal pressure measured by the guiding catheter (Pa). For subtotal or chronically occluded arteries, a default FFR value of 0.50 will be assigned to the vessel, in accordance with prior multicenter trials [3]. Cineangiographic images and FFR measurements will be recorded for “integration” with the CCTA and FFR_CT_ measurements.

Patients who undergo PCI by intracoronary stenting of ≥1 coronary lesions after the ICA and FFR measurements may have repeat measurements of FFR after the completion of stent deployment, as clinically indicated. Post-stent FFR measurements will be performed in a manner similar to pre-PCI FFR, with continuous pressure recordings and angiographic images of the position of the pressure wire captured along with the measured FFR.

**References - Online Supplementary Material**

1. Smith SC, Jr., Feldman TE, Hirshfeld JW, Jr., Jacobs AK, Kern MJ, King SB, 3rd et al. ACC/AHA/SCAI 2005 guideline update for percutaneous coronary intervention: a report of the American College of Cardiology/American Heart Association Task Force on Practice Guidelines (ACC/AHA/SCAI Writing Committee to Update the 2001 Guidelines for Percutaneous Coronary Intervention). J Am Coll Cardiol. 2006;47(1):e1-121. doi:10.1016/j.jacc.2005.12.001.

2. Raff GL, Abidov A, Achenbach S, Berman DS, Boxt LM, Budoff MJ et al. SCCT guidelines for the interpretation and reporting of coronary computed tomographic angiography. J Cardiovasc Comput Tomogr. 2009;3(2):122-36. doi:10.1016/j.jcct.2009.01.001.

3. Tonino PA, De Bruyne B, Pijls NH, Siebert U, Ikeno F, van' t Veer M et al. Fractional flow reserve versus angiography for guiding percutaneous coronary intervention. N Engl J Med. 2009;360(3):213-24. doi:10.1056/NEJMoa0807611.
